# A Thrombomodulin Promoter Gene Polymorphism, rs2239562, Influences Both Susceptibility to and Outcome of Sepsis

**DOI:** 10.3389/fmed.2021.762198

**Published:** 2022-01-10

**Authors:** Eizo Watanabe, Osamu Takasu, Youichi Teratake, Teruo Sakamoto, Toshiaki Ikeda, Joji Kotani, Nobuya Kitamura, Masaaki Ohmori, Ayako Teratani, Goichi Honda, Masahiko Hatano, Benjamin Mayer, E. Marion Schneider, Shigeto Oda

**Affiliations:** ^1^Department of Emergency and Critical Care Medicine, Chiba University Graduate School of Medicine, Chiba, Japan; ^2^General Medical Science, Chiba University Graduate School of Medicine, Chiba, Japan; ^3^Department of Emergency and Critical Care Medicine, Kurume University School of Medicine, Kurume, Japan; ^4^Biomedical Research Center, Chiba University, Chiba, Japan; ^5^Division of Critical Care and Emergency Medicine, Tokyo Medical University Hachioji Medical Center, Hachioji, Japan; ^6^Department of Emergency, Disaster and Critical Care Medicine, Hyogo College of Medicine, Nishinomiya, Japan; ^7^Department of Emergency and Critical Care Medicine, Kimitsu Chuo Hospital, Kisarazu, Japan; ^8^Medical Affairs Department, Asahi Kasei Pharma Corporation, Tokyo, Japan; ^9^Institute for Epidemiology and Medical Biometry, Ulm Universtiy, Ulm, Germany; ^10^Division of Experimental Anesthesiology, University Hospital Ulm, Ulm, Germany

**Keywords:** genetic predisposition to disease, genetic testing, multicenter studies, disseminated intravascular coagulation, single nucleotide polymorphisms

## Abstract

**Objective:** Disseminated intravascular coagulation plays a key role in the pathophysiology of sepsis. Thrombomodulin is essential in the protein C system of coagulation cascade, and functional polymorphisms influence the human thrombomodulin gene (*THBD*). Therefore, we conducted a multicenter study to evaluate the influence of such polymorphisms on the pathophysiology of sepsis.

**Methods:** A collaborative case-control study in the intensive care unit (ICU) of each of five tertiary emergency centers. The study included 259 patients (of whom 125 displayed severe sepsis), who were admitted to the ICU of Chiba University Hospital, Chiba, Japan between October 2001 and September 2008 (discovery cohort) and 793 patients (of whom 271 patients displayed severe sepsis), who were admitted to the five ICUs between October 2008 and September 2012 (multicenter validation cohort). To assess the susceptibility to severe sepsis, we further selected 222 critically ill patients from the validation cohort matched for age, gender, morbidity, and severity with the patients with severe sepsis, but without any evidence of sepsis.

**Results:** We examined whether the eight *THBD* single nucleotide polymorphisms (SNPs) were associated with susceptibility to and/or mortality of sepsis. Higher mortality on severe sepsis in the discovery and combined cohorts was significantly associated with the CC genotype in a *THBD* promoter SNP (−1920^*^C/G; rs2239562) [odds ratio [*OR*] 2.709 (1.067–6.877), *P* = 0.033 and *OR* 1.768 (1.060–2.949), *P* = 0.028]. Furthermore, rs2239562 SNP was associated with susceptibility to severe sepsis [*OR* 1.593 (1.086–2.338), *P* = 0.017].

**Conclusions:** The data demonstrate that rs2239562, the *THBD* promoter SNP influences both the outcome and susceptibility to severe sepsis.

## Introductions

Sepsis is a global public death emergency, affecting millions of people worldwide, and representing one of the greatest global causes of mortality (1). Currently, numerous genetic polymorphisms are suggested to be associated with susceptibility to and/or outcome of sepsis, and we discovered several polymorphisms related to sepsis pathophysiology (2). One of the purposes of investigating the effects of genetic polymorphisms on the clinical course of diseases is to examine the association of particular molecular pathways, that is, cytokine networks (3, 4), cell death (5, 6), and coagulation/fibrinolysis systems among others (7–9).

Recently, precision medicine has gained attention, particularly for multifactorial diseases in the critical care field, and disseminated intravascular coagulation (DIC) in the pathophysiology of sepsis has of late been increasingly recognized to play a key role as well as to be a therapeutic target (10). Thrombomodulin is an integral membrane protein expressed on the surface of endothelial cells and serves as a cofactor for thrombin, having a pivotal role in the protein C system through the extracellular domain, which binds to thrombin. Thereby, thrombomodulin activates protein C and prevents excessive coagulation (11). Therefore, the recombinant agent is thought to be a promising drug for sepsis-induced coagulopathy (12–14). A recent clinical trial using recombinant human thrombomodulin (ART-123) still showed a tendency of survival benefit in phase three multicenter study (14). Activated protein C (APC) decomposes the coagulation factors Va and VIIIa, thus exerting the anti-coagulative properties (15). The pathophysiology of sepsis-induced DIC is recognized as a perfusion abnormality by fibrin clotting, against which APC has therapeutic potential. Additionally, APC acts both by activating the endothelial receptors, such as protease-activated receptor-1and endothelial protein C receptor, as well as by degrading histones (16). Although the polymorphisms of protein C genes, for example, PROC(−1641), are already demonstrated to be associated with the mortality and organ failures of sepsis (9), there is only limited investigation of the thrombomodulin gene *THBD*.

Accordingly, we postulated that some of the *THBD* single nucleotide polymorphisms (SNPs) are associated with susceptibility to and/or mortality of sepsis. In addition, the present study aimed to evaluate the role of thrombomodulin in the pathophysiology of sepsis through a genetic association study with Japanese multicenter cohorts, focusing on the *THBD* gene polymorphisms.

## Materials and Methods

### Patient Selection

**Figure 1:**
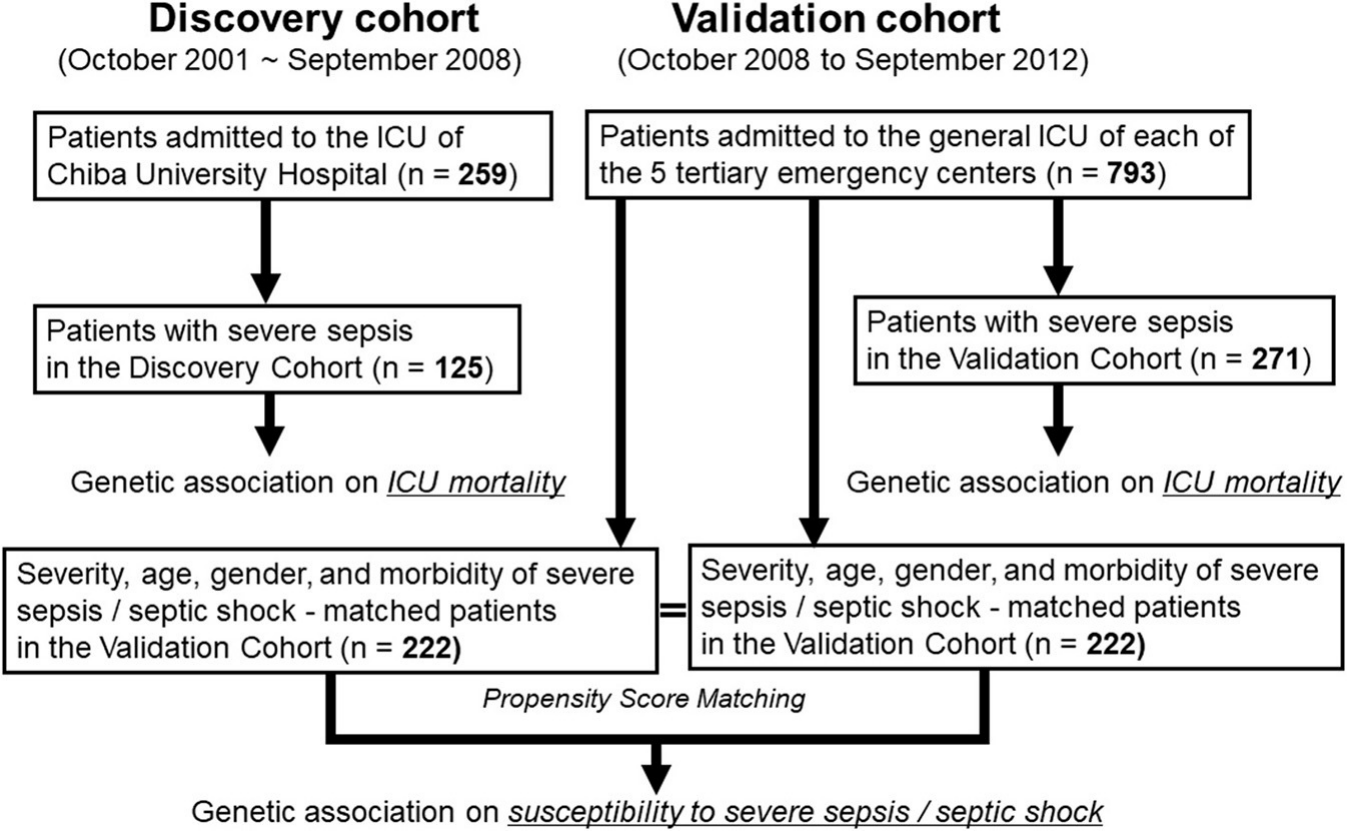
Flow diagram of patient inclusion.

The subjects were recruited as part of a prospective, observational study of adults admitted during 2001–2012 into a network of Japanese intensive care units (ICUs). The study using these subjects has been reported elsewhere (6). The protocol was approved by the institutional Ethics Committees at all the five participating institutes [the Ethics Committee of Chiba University School of Medicine (permission number 205), the Ethical Committee of Kurume University (bioethics permission number 49), the Medical Research Ethics Committee of Tokyo Medical University, the Ethics Review Board of Hyogo College of Medicine (permission number 208), and the Ethics Committee of Kimitsu Chuo Hospital (permission number 120)]. Following approval by the institutional ethics committees, a written informed consent was obtained from the patients or their next of kin. Figure 1 summarizes the patient inclusion process.

### Discovery Cohort

In total, 259 critically ill patients admitted to the ICU of Chiba university hospital in Chiba, Japan, between October 2001 and September 2008 were included (Table 1). The inclusion criteria were as follows: admission to the ICU, aged 20 years or older, and the patients able to provide informed written consent or obtainable from a family member or the legal representative. The exclusion criteria are as follows: pregnancy, treatment for hematologic malignancies, the patients receiving radiation treatment and chemotherapy, a history of genetic therapy, and being outside the scope of active treatment. The blood samples were obtained immediately after admission to the ICU. The genomic DNA was extracted from the whole blood cells.

**Table 1:** Baseline characteristics of the study population.

	**Discovery cohort**				**Multicenter validation cohort**			
	**All *n* = 259**	**Non-sepsis patients *n* = 104**	**SS patients *n* = 125**	***P***	**All *n* = 793**	**Non-sepsis patients *n* = 454**	**SS patients *n* = 271**	***P***
Age (years), mean ± SD	56.8 ± 17.4	56.5 ± 17.5	57.6 ± 17.3	0.492^*^	63.9 ± 17	62.5 ± 17.9	66.6 ± 15.0	0.0008^*^
Male/female gender, *n*	146/113	60/44	69/56	0.659^**^	517/276	296/158	176/95	0.915^**^
Length of ICU stay (days), median (IQR)	6 (3–15)	3 (1–7)	13 (7–15)	<0.0001^*^	9 (4–19)	7 (4–14)	15 (7–29.3)	<0.0001^*^
SOFA score, median (IQR)	6 (3–11)	3 (1–6)	10 (7–13)	<0.0001^*^	6 (3–9)	4 (2–6)	9 (6–12)	<0.0001^*^
APACHE II score, median (IQR)	15 (9.3–22)	10 (6–14)	21 (17–27)	<0.0001^*^	16 (10–23)	13 (8–19.8)	21 (16–26)	<0.0001^*^
Severe sepsis morbidity (%)	48.3				33.8			
Mortality (%)	17	5.71	29.6	<0.0001^**^	12.2	5.4	25.3	<0.0001^**^
**Post-surgical operation**								
Post-cardiovascular surgery, *n* (%)	28 (10.8)	24 (23.0)	4 (3.2)	<0.0001^**^	21 (2.6)	5 (1.1)	12 (4.4)	0.009^**^
Post-gastrointestinal surgery, *n* (%)	33 (12.7)	11 (10.6)	15 (12.0)	0.835^**^	79 (10.0)	25 (5.5)	43 (15.9)	<0.0001^**^
Others, *n* (%)	20 (7.7)	12 (11.5)	6 (4.8)	0.083^**^	6 (0.8)	3 (0.7)	3 (1.1)	0.677^**^
Intracranial disease (ICH/CI), *n* (%)	5 (2.0)	0 (0)	4 (3.2)	0.128^**^	68 (8.6)	62 (13.7)	3 (1.1)	<0.0001^**^
Respiratory failure, *n* (%)	28 (10.8)	4 (3.8)	22 (17.6)	0.001^**^	77 (9.7)	15 (3.3)	51 (18.8)	<0.0001^**^
Heart failure, *n* (%)	10 (3.9)	4 (3.8)	3 (2.4)	0.705^**^	72 (9.1)	61 (13.4)	8 (3.0)	<0.0001^**^
**Endogenous abdominal disease**								
Acute pancreatitis, *n* (%)	22 (8.5)	16 (15.4)	6 (4.8)	0.012^**^	39 (4.9)	22 (4.8)	14 (5.2)	0.861^**^
Gastrointestinal bleeding, *n* (%)	5 (2.0)	2 (1.9)	3 (2.4)	>0.9999^**^	36 (4.5)	33 (7.3)	2 (0.7)	<0.0001^**^
Hepatic failure, *n* (%)	7 (2.7)	3 (2.9)	4 (3.2)	>0.9999^**^	19 (2.4)	7 (1.5)	9 (3.3)	0.124^**^
Others, *n* (%)	13 (5.0)	1 (1.0)	9 (7.2)	0.024^**^	34 (4.3)	10 (2.2)	21 (7.7)	0.001^**^
CPAOA, *n* (%)	4 (1.5)	2 (1.9)	2 (1.6)	>0.9999^**^	39 (4.9)	32 (7.0)	4 (1.5)	0.001^**^
Trauma, *n* (%)	11 (4.3)	9 (8.7)	0 (0)	0.001^**^	119 (15.0)	105 (23.1)	8 (3.0)	<0.0001^**^
Intoxication, *n* (%)	8 (3.1)	4 (3.8)	4 (3.2)	>0.9999^**^	23 (2.9)	17 (3.7)	6 (2.2)	0.283^**^
Burn, *n* (%)	2 (0.8)	1 (1.0)	0 (0)	0.454^**^	12 (1.5)	7 (1.5)	4 (1.5)	>0.9999^**^
Others, *n* (%)	63 (24.3)	11 (10.6)	43 (34.4)	<0.0001^**^	149 (18.8)	50 (11.0)	83 (30.6)	<0.0001^**^

P values (non-sepsis patients vs. severe sepsis/septic shock patients) were calculated with Student's t-test or Mann-Whitney U-test^*^ and Fisher's exact test^**^.

*ICH, intracranial hemorrhage; CI, cerebral infarction; CPAOA, cardiopulmonary arrest on arrival; SS, severe sepsis/septic shock; SOFA, sequential organ failure assessment; APACHE II, the acute physiology and chronic health evaluation; SD, standard deviation, IQR, interquartile range. Discovery and validation cohorts include 30 and 68 mild sepsis patients (non-severe), respectively*.

### Multicenter Validation Cohort

In the multicenter validation cohort, 793 critically ill patients admitted to the general ICU of each of the five tertiary emergency centers of Kurume University Hospital, Tokyo Medical University Hachioji Medical Center, Hyogo College of Medicine, Kimitsu Chuo Hospital, and Chiba University Hospital (updated permission number 457) from October 2008 to September 2012 were included (Table 1). The inclusion and exclusion criteria were the same as for the discovery cohort. The blood cells were refrigerated and transferred to the Chiba University Hospital, where the genomic DNA was subsequently extracted.

### Propensity Score Matching in Multicenter Cohort

We conducted a case-cohort study to compare the *THBD* genotypic distributions in whole blood genomic DNA from the critically ill patients with severe sepsis vs. non-sepsis with similar age, gender, severity of illness, and mortality to assess the genetic association for susceptibility to sepsis between the similar severity of critically ill patients. The patients with sepsis tend to die than the patients with non-sepsis do. Therefore, propensity score matching was implemented to compare *THBD* genotypic distributions between the severe sepsis and non-sepsis having similar severity, such as mortality. Controls were matched as follows: the participating centers submitted similar blood samples from critically ill patients who were not known to have sepsis. All the patients who did not meet severe sepsis criteria were placed into the matching pool, and those who had any evidence of sepsis were then excluded. Each patient with sepsis was tentatively matched with all the patients in the pool of the same gender and hospital discharge status (alive vs. dead). Then, each patient with severe sepsis was matched with one patient from its set of potential non-sepsis matches. The patient selection above was performed employing the propensity score matching method with a Greedy 5-to-1 digit-matching algorithm for the clinical factors, that is, age, gender, severity scores, and hospital discharge status. Once all the propensity-score matching was performed, we compared the baseline covariates between the two groups. Ultimately, 444 patients (222 with severe sepsis/septic shock (SS) while 222 displayed non-sepsis) were selected (Table 2).

**Table 2:** Propensity score-matched patients with severe sepsis and without any evidence of sepsis in the validation cohort (post-matching results).

	**Non-sepsis patients *n* = 222**	**SS patients *n* = 222**
Age (years), mean ± SD	66.7 ± 16.4	66.2 ± 14.9
Male/female gender, *n*	194/222	191/222
SOFA score, mean ± SD	6.6 ± 3.7	9.1 ± 4.1
APACHE II score, mean ± SD	21.0 ± 7.7	20.8 ± 7.6
Mortality (%)	35.4	35.9

*SS, severe sepsis/septic shock; SOFA, sequential organ failure assessment; APACHE II, the acute physiology and chronic health evaluation; SD, standard deviation*.

### Data Collection

The baseline characteristics (age and gender) and clinical data, such as length of ICU stay, Sequential Organ Failure Assessment (SOFA) scores (17), Acute Physiology and Chronic Health Evaluation (APACHE) II scores (18), morbidity of severe sepsis and septic shock, and ICU mortality, were obtained after the patients were documented at study entry. The APACHE II scores and SOFA scores were calculated during the first 24 h after admission. The diagnosis of sepsis, severe sepsis, and septic shock was based on the criteria presented at the American College of Chest Physicians/Society of Critical Care Medicine Consensus Conference in 1992 (19). In the present study, both the severe sepsis and septic shock are expressed together as the SS group.

### SNP Selection and Genotyping

Genomic DNA was isolated from the banked whole blood specimens collected on ICU admission. We genotyped eight markers from the region surrounding *THBD* (Supplementary Table 1). Genotyping of SNPs was performed using the APEX testing methods, an outsourced service provided by Asper Biogene (Tartu, Estonia, https://www.asperbio.com/, accessed on 2021/5/25). Eight SNPs distributed in *THBD* and its promoter region (rs2239562, rs3216183, rs121918667, rs1800577, rs1042579, rs41348347, rs1042580, and rs3176123) were investigated. We were able to assign the genotype in >95% of typed samples. To verify the genotypes of the SNPs, some were tested in a set of earlier Japanese samples (6). The allelic statuses were determined employing the PCR with sequence-specific primers, using the TaqMan^®^ probe (Applied Biosystems, Foster City, CA, USA). The genotyped SNPs can be found at dbSNP (http://www.ncbi.nlm.nih.gov/SNP/). The SNP genotyping matching rate was 100%, according to the earlier results (6).

### Statistical Analysis

The primary endpoint was a mortality of severe sepsis, and the secondary endpoint was susceptibility to severe sepsis concerning the *THBD* SNP. Hardy–Weinberg equilibrium (HWE) for the population distribution of the variant alleles was determined according to the approach described by Guo and Thompson (20). The allelic *chi*-square tests were applied for each SNP. The statistical analyses for the genetic association tests were performed using the SNP & Variation Suite 8.8.3 software (Golden Helix, Bozeman, MT, USA). Significant differences in the mean ± SD or median (interquartile range; IQR) values between the two groups were evaluated by using the Student's *t*-test or Mann–Whitney *U*-test, depending on the variables. The statistical analyses were performed using the GraphPad PRISM 8, version 8.3.0 (GraphPad Software, San Diego, CA, USA) for Windows. We considered differences to be significant at *P* <.05.

## Results

### The Baseline Characteristics of the Discovery and Multicenter Validation Cohorts

Table 1 summarizes the baseline characteristics of the discovery cohort (*n* = 259) and the multicenter validation cohort (*n* = 793). Mortality and the SOFA and APACHE II scores were significantly higher in the SS group than those in the non-sepsis group in both the cohorts (*P* < 0.0001, Table 1). In the validation cohort, the SS patients were significantly older than the non-septic controls (*P* = 0.0008, Table 1). By contrast, no significant difference in the SOFA or APACHE II scores was detectable between the SS groups of the two cohorts (*P* = 0.1126, 0.8714, respectively), which indicated that there was no marked difference in severity in the SS patients between the two cohorts. In the validation cohort, trauma, heart failure, and intracranial diseases were overrepresented in the non-sepsis group.

### Genotype Distributions of Eight *THBD* SNPs Related to the Outcome of Severe Sepsis

To determine the frequencies of eight SNPs of *THBD*, which are associated with the mortality of severe sepsis, genotyping of these polymorphisms was performed in the discovery cohort. The observed allele frequencies and genotypic distributions for the investigated polymorphisms are summarized in Supplementary Table 2 (discovery cohort), **3** (validation cohort), and **4** (combined cohort). The distributions of genotypes for all the investigated polymorphisms conformed to the HWE test (*P* > 0.01).

**Figure 2:**
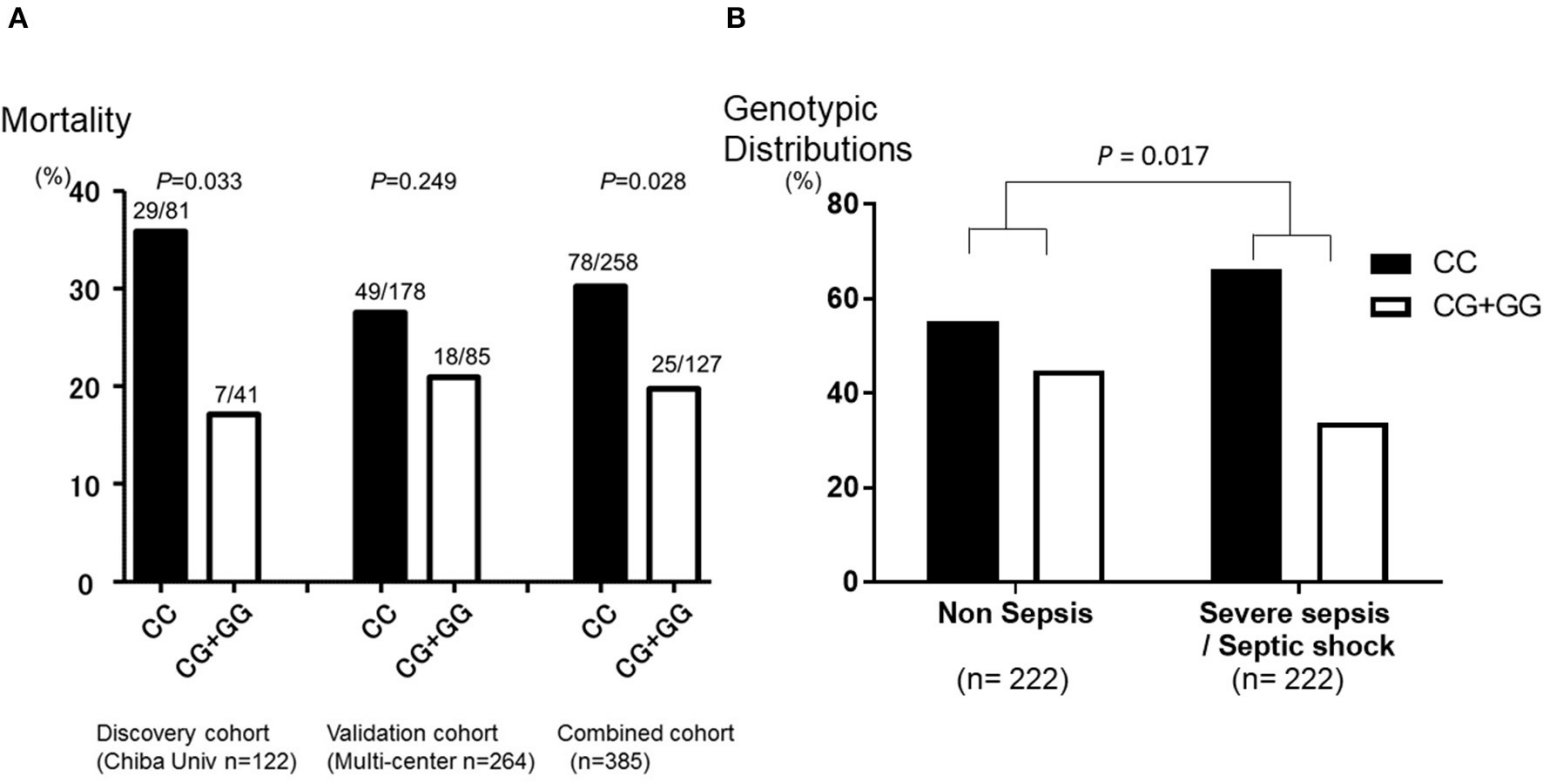
Mortality and morbidity of severe sepsis in genotype categories of *thrombomodulin gene (THBD)* single nucleotide polymorphisms (SNPs) rs2239562. **(A)** The *Y*-axis of the graph shows the mortality of severe sepsis in the SNPs that is in the promoter region of *THBD* (−1920^*^C/G; rs2239562). The patients with the CC genotype of rs2239562 were significantly associated with worse outcome of severe sepsis than the CG + GG genotype of the SNP in the discovery cohort (*P* = 0.033). The trend was maintained in the validation cohort (*P* = 0.249), and the association was strengthened in the combined cohort (*P* = 0.028). **(B)** The *Y*-axis of the graph shows the genotypic distributions of the SNP that is in the promoter region of *THBD* (−1920^*^C/G; rs2239562) in the patients with severe sepsis and without any evidence of sepsis (non-sepsis). The percentage of patients with the CC genotype of rs2239562 was significantly higher in the SS group than those in the non-sepsis group (*P* = 0.017). *The P* values for the SNP were evaluated with the chi-square test on the dominant model analysis with the correlation/trend test.

The genotype call rate of the eight SNPs was 96.3–100%, although the genotypic distributions in rs1042579 diverged from the HWE in the studied subjects (*P* = 0.005, Supplementary Table 4). Among the eight SNPs, a *THBD* promoter SNP (−1920^*^C/G; rs2239562) was found to be most significantly associated with mortality of SS group in the discovery cohort [odds ratio (*OR*) 2.709 (range of lower and upper confidence bound; 1.067–6.877), *P* = 0.033 in a dominant model with the trend test, Figure 2A] and the trend was maintained in the validation cohort [*OR* 1.446 (0.782–2.675), *P* = 0.249, Figure 2A]. As a result, the association was statistically strengthened in the combined cohort [*OR* 1.768 (1.060–2.949), *P* = 0.028 in a dominant model with the trend test, Figure 2A]. Consequently, the carriage of the CC genotype was significantly associated with a worse outcome of SS group. All the genotypic distributions in relation to a mortality of SS of both the cohorts are presented in Supplementary Tables 2–4.

### Genotypic Distributions of Eight *THBD* SNPs Related to Susceptibility to Severe Sepsis

Because of the retrospective nature of the study, the baseline imbalances between the SS and non-sepsis existed; therefore, we identified 222 subjects from each of the SS and non-sepsis groups from the validation cohort by propensity score matching (17) to equalize morbidity of severe sepsis and severity of illness of both the cohorts (Table 2). Figure 1 summarizes the patient inclusion process. By matching with the Propensity Score in the multicenter validation cohort, 222 patients with severe sepsis and the same number of controls with a similar number of ICU deaths, the severity of illness (APACHEII), and age and gender-matched without evidence of any infection also admitted to the ICUs were included (Table 2). The genotypic distributions of the *THBD* promoter SNP (rs2239562) and the *THBD* exon 1 SNP (rs41348347) were significantly different between the SS and non-sepsis groups with similar severity of illness [*OR* 1.593 (1.086–2.338), *P* = 0.017 and *OR* 0.107 (0.013–0.853), *P* = 0.011, respectively in a dominant model with the trend test, Figure 2B]. By contrast, the minor allele frequency of the exon SNP rs41348347 was too low to reveal the clinical implications (1.14%), and this SNP was found not to be related to the outcome of SS patients in the previous analysis (Supplementary Tables 2–4). Therefore, the association between rs2239562 and susceptibility to SS was confirmed, even though both the groups were similar for severity and the other background characteristics. The genotype call rate of the eight SNPs was 93.9–100%, and the genotypic distributions in all the eight SNPs did not diverge from the HWE in the studied subjects (Supplementary Table 5).

## Discussions

**Figure 3:**
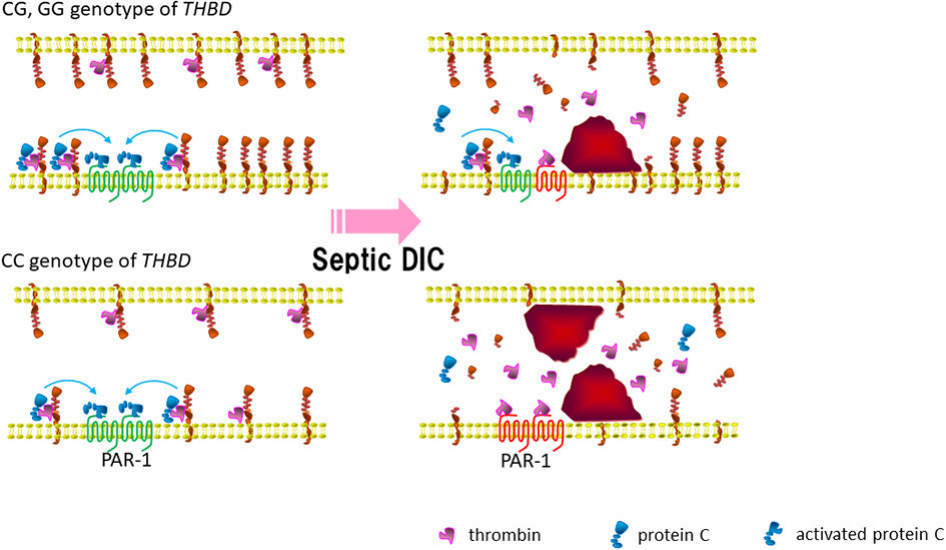
*Thrombomodulin gene* promotor may influence thrombomodulin guided activated protein C (APC) function and limit endothelial cell damage in sepsis. In the blood stream, the thrombomodulin levels influence APC and antiinflammatory PAR-1 signaling. Bottom right: restricted thrombomodulin (due to carriage of CC genotype of rs2239562) promote thrombin induced inflammation and endothelial cell damage. APC, activated protein C; PAR-1, protease-activated receptor-1; DIC, disseminated intravascular coagulation.

The coagulation disorders are common in sepsis, and the patients frequently progress to develop DIC. The present study indicated that a coagulopathy-associated *THBD* promoter SNP, rs2239562, had a significant influence on the outcome as well as the progress of severe sepsis/septic shock. First, we determined the worse outcome of severe sepsis to be associated with the CC (major) allele homozygotes of rs2239562. Intriguingly, the frequency of the CC carriers was also augmented in the SS in a propensity-matched patient cohort. In the pathophysiology of sepsis, *THBD* promotor may influence the thrombomodulin-guided APC function and limit endothelial cell damage (Figure 3). In the bloodstream, the thrombomodulin levels influence APC and antiinflammatory protease-activated receptor-1 (PAR-1) signaling. The restricted thrombomodulin (due to carriage of CC genotype of rs2239562) promotes thrombin-induced inflammation and endothelial cell damage.

Some drugs in development aim to regulate sepsis-induced coagulopathy or when inflammation has been initiated. One of the promising drugs is ART-123, a recombinant form of the anticoagulant protein thrombomodulin from Asahi Kasei Pharma that is currently in a global clinical trial (14, 21). Emerging evidence is accumulating, which demonstrates the therapeutic efficacy of ART-123 (22, 23), and the mechanisms of thrombomodulin of not only anticoagulation but also anti-inflammation through the adsorption of high-mobility group-B1 (HMG-B1) have been advocated (24). In the recently published Japanese Guidelines, ART-123 has been weakly recommended for the patients with sepsis with DIC (25). From a practical point, the proper use of ART-123, such as target-illness severity and dosage for the patients with sepsis-induced DIC, remains unclear, particularly for those with renal impairment (26) as well as for those on continuous hemodiafiltration (27). Because of the anticoagulant properties of ART-123, the most critical concern in treating the patients with DIC, who are susceptible to bleeding, is a severe hemorrhage due to abrupt increases in plasma concentration of this drug. The present study results should shed light on the precision medicine for sepsis-induced DIC utilizing the pharmacogenetics associated with the coagulation system.

An earlier study reported soluble thrombomodulin concentrations and deep venous thrombosis associated with 2729A>C and A455V missense mutations in the Japanese (28). The *THBD* 1418T allele in rs1042579 SNP was associated with the lower soluble thrombomodulin levels, both in plasma and in HUVEC-conditioned medium, and with an increase in functional membrane-bound thrombomodulin in HUVEC, explaining the increased APC levels and the reduced venous thromboembolism risk (29). Interestingly, *THBD* is also recognized as a pathogenic gene of the atypical hemolytic uremic syndrome (aHUS) (30), caused by complement dysregulation and may occasionally be triggered by a septic insult. Therefore, the present data indicating that a *THBD* polymorphism influenced the susceptibility, as well as the outcome of sepsis, might be key in the pathogenesis of aHUS. To counteract sepsis-triggered aHUS, the continued accumulation of knowledge regarding the patterns of disease onset and response to the treatments under different genetic backgrounds, such as *THBD*, will be essential for developing future treatment strategies (31). Further, a recent study demonstrated that one of the *THBD* SNPs, rs1962, was related to the risk of death in the patients with sepsis (32). The above reports support the deep association between the *THBD* SNPs and the pathophysiology of sepsis-induced organ dysfunctions.

Our work has several limitations. First, we used the criteria of the 2013 surviving sepsis campaign guidelines to include patients because this work was initiated before introducing the SEPSIS-3 diagnostic criteria (33). Second, the statistical significance of the results of the *THBD* genetic association was relatively weak because the patient characteristics were very heterogeneous, such that it was challenging to show the influence on an outcome with the present sample volume. Interestingly, a recent study demonstrates that phenotype γ and δ reveals the characteristics of hematologic dysfunctions, such as coagulopathy (34). A survey in Japan conducted by the Japanese Association for Acute Medicine reported that the incidence of DIC is high and exceeded 50% in sepsis (35). At least in part, these may explain the lack of significance concerning the association between the *THBD* SNP and the incidence of SS in “the less severe” validation cohort. More studies along these lines will clarify these questions. Third, neither the gene expression nor biomarker values can be recruited since we performed piggyback evaluation using the DNA samples from an earlier genetic association study of sepsis (6). Ideally, we might have examined the concentrations of soluble thrombomodulin as a phenotype parameter of the rs2239652 SNP promotor influence. However, it is also true that the concentrations do not always correlate with sepsis severity (36). Finally, any data on site of infection, administered antimicrobials, microbiology, and coagulopathy markers were not provided. Although we used a prospective registry of the genetic association study for the critically ill patients, the data were not mandatory in the long-continued cohorts. Even in such miscellaneous populations, the rs2239562 *THBD* SNP was associated with susceptibility to and outcome of SS. This suggests the crucial role of the coagulation system in the pathophysiology of sepsis.

## Conclusions

Our mid-scale population association study supports the hypothesis that the genetic predispositions to severe sepsis as well as to the worse outcome of sepsis exist. Consequently, a variation in the promoter region of the *THBD* appears to explain, in part, the susceptibility to severe sepsis/septic shock in the Japanese multicenter ICU. Whole-genome sequencing targeting *THBD* with a more extensive study population is warranted to be able to transfer the present data to the clinical settings.

## Data Availability Statement

The datasets presented in this study can be found in online repositories. The names of the repository/repositories and accession number(s) can be found in the article/Supplementary Material.

## Ethics Statement

The studies involving human participants were reviewed and approved by the Ethics Committee of Chiba University School of Medicine (permission number 205), the Ethical Committee of Kurume University (bioethics permission number 49), the Medical Research Ethics Committee of Tokyo Medical University, the Ethics Review Board of Hyogo College of Medicine (permission number 208), and the Ethics Committee of Kimitsu Chuo Hospital (permission number 120). The patients/participants provided their written informed consent to participate in this study.

## Author Contributions

EW, MH, and SO: conceived and designed the experiments. YT, MO, and AT: performed the experiments. EW, YT, MO, BM, and MH: analyzed the data. OT, TS, TI, JK, NK, and EMS: contributed reagents, materials, and analysis tools. EW and GH: wrote the paper. All authors contributed to the article and approved the submitted version.

## Funding

The present study is partly funded by Asahi Kasei Pharma Corporation, which had no influence on the results of the present study (EW). Funded also by A-STEP (Adaptive and Seamless Technology transfer Program) through targetdriven R&D; AS251Z02001P) (EW). MO was the recipient of a Therapeutics Research Initiative Grant from Chiba University School of Medicine 2018-S3.

## Conflict of Interest

The authors declare that the research was conducted in the absence of any commercial or financial relationships that could be construed as a potential conflict of interest.

## Publisher's Note

All claims expressed in this article are solely those of the authors and do not necessarily represent those of their affiliated organizations, or those of the publisher, the editors and the reviewers. Any product that may be evaluated in this article, or claim that may be made by its manufacturer, is not guaranteed or endorsed by the publisher.

## References

[1] Fleischmann C Scherag A Adhikari NK Hartog CS Tsaganos T Schlattmann P. International forum of acute care T: assessment of global incidence and mortality of hospital-treated sepsis. Current estimates and limitations. Am J Respir Crit Care Med. (2016) 193:259–72. 10.1164/rccm.201504-0781OC 26414292

[2] Cohen J Vincent JL Adhikari NK Machado FR Angus DC Calandra T. Sepsis: a roadmap for future research. Lancet Infect Dis. (2015) 15:581–614. 10.1016/S1473-3099(15)70112-X 25932591

[3] Watanabe E Hirasawa H Oda S Matsuda K Hatano M Tokuhisa T. Extremely high interleukin-6 blood levels and outcome in the critically ill are associated with tumor necrosis factor- and interleukin-1-related gene polymorphisms. Crit Care Med. (2005) 33:89–97; discussion 242–243. 10.1097/01.CCM.0000150025.79100.7D 15644653

[4] Watanabe E Buchman TG Hirasawa H Zehnbauer BA. Association between lymphotoxin-alpha (tumor necrosis factor-beta) intron polymorphism and predisposition to severe sepsis is modified by gender and age. Crit Care Med. (2010) 38:181–93. 10.1097/CCM.0b013e3181bc805d 19789445 PMC5124381

[5] Frank AJ Sheu CC Zhao Y Chen F Su L Gong MN. BCL2 genetic variants are associated with acute kidney injury in septic shock^*^. Crit Care Med. (2012) 40:2116–23. 10.1097/CCM.0b013e3182514bca 22710204 PMC3690367

[6] Kimura T Watanabe E Sakamoto T Takasu O Ikeda T Ikeda K. Autophagy-related IRGM polymorphism is associated with mortality of patients with severe sepsis. PLoS ONE. (2014) 9:e91522. 10.1371/journal.pone.0091522 24626347 PMC3953488

[7] Haralambous E Hibberd ML Hermans PW Ninis N Nadel S Levin M. Role of functional plasminogen-activator-inhibitor-1 4G/5G promoter polymorphism in susceptibility, severity, and outcome of meningococcal disease in Caucasian children. Crit Care Med. (2003) 31:2788–93. 10.1097/01.CCM.0000100122.57249.5D 14668616

[8] Madach K Aladzsity I Szilagyi A Fust G Gal J Penzes I. 4G/5G polymorphism of PAI-1 gene is associated with multiple organ dysfunction and septic shock in pneumonia induced severe sepsis: prospective, observational, genetic study. Crit Care. (2010) 14:R79. 10.1186/cc8992 20429897 PMC2887202

[9] Walley KR Russell JA. Protein C−1641 AA is associated with decreased survival and more organ dysfunction in severe sepsis. Crit Care Med. (2007) 35:12–7. 10.1097/01.CCM.0000249823.44726.4E 17080006

[10] Vincent JL Castro P Hunt BJ Jorres A Praga M Rojas-Suarez J. Thrombocytopenia in the ICU: disseminated intravascular coagulation and thrombotic microangiopathies-what intensivists need to know. Crit Care. (2018) 22:158. 10.1186/s13054-018-2073-2 29895296 PMC5998546

[11] Mohri M Sugimoto E Sata M Asano T. The inhibitory effect of recombinant human soluble thrombomodulin on initiation and extension of coagulation–a comparison with other anticoagulants. Thromb Haemost. (1999) 82:1687–93. 10.1055/s-0037-1614900 10613656

[12] Vincent JL Ramesh MK Ernest D LaRosa SP Pachl J Aikawa N. A randomized, double-blind, placebo-controlled, Phase 2b study to evaluate the safety and efficacy of recombinant human soluble thrombomodulin, ART-123, in patients with sepsis and suspected disseminated intravascular coagulation. Crit Care Med. (2013) 41:2069–79. 10.1097/CCM.0b013e31828e9b03 23979365

[13] Yoshihiro S Sakuraya M Hayakawa M Ono K Hirata A Takaba A. Recombinant human soluble thrombomodulin contributes to reduced mortality in sepsis patients with severe respiratory failure: a retrospective observational study using a multicenter dataset. Shock. (2018) 51:174–9. 10.1097/SHK.0000000000001148 29596106 PMC6319596

[14] Vincent JL Francois B Zabolotskikh I Daga MK Lascarrou JB Kirov MY. Effect of a recombinant human soluble thrombomodulin on mortality in patients with sepsis-associated coagulopathy: the SCARLET randomized clinical trial. JAMA. (2019) 321:1993–2002. 10.1001/jama.2019.5358 31104069 PMC6547077

[15] Schouten M Wiersinga WJ Levi M van der Poll T. Inflammation, endothelium, and coagulation in sepsis. J Leukoc Biol. (2008) 83:536–45. 10.1189/jlb.0607373 18032692

[16] Chaput C Zychlinsky A. Sepsis: the dark side of histones. Nat Med. (2009) 15:1245–6. 10.1038/nm1109-1245 19893552

[17] Vincent JL de Mendonca A Cantraine F Moreno R Takala J Suter PM. Use of the SOFA score to assess the incidence of organ dysfunction/failure in intensive care units: results of a multicenter, prospective study. Working group on “sepsis-related problems” of the European Society of Intensive Care Medicine. Crit Care Med. (1998) 26:1793–800. 10.1097/00003246-199811000-00016 9824069

[18] Knaus WA Draper EA Wagner DP Zimmerman JE. APACHE II: a severity of disease classification system. Crit Care Med. (1985) 13:818–29. 10.1097/00003246-198510000-00009 3928249

[19] American College of Chest Physicians/Society of Critical Care Medicine Consensus Conference: definitions for sepsis and organ failure and guidelines for the use of innovative therapies in sepsis. Crit Care Med. (1992) 20:864–74. 10.1097/00003246-199206000-00025 1597042

[20] Guo SW Thompson EA. Performing the exact test of Hardy-Weinberg proportion for multiple alleles. Biometrics. (1992) 48:361–72. 10.2307/2532296 1637966

[21] Williams SC. After Xigris, researchers look to new targets to combat sepsis. Nat Med. (2012) 18:1001. 10.1038/nm0712-1001 22772543

[22] Yoshimura J Yamakawa K Ogura H Umemura Y Takahashi H Morikawa M. Benefit profile of recombinant human soluble thrombomodulin in sepsis-induced disseminated intravascular coagulation: a multicenter propensity score analysis. Crit Care. (2015) 19:78. 10.1186/s13054-015-0810-3 25883031 PMC4367899

[23] Yamakawa K Levy JH Iba T. Recombinant human soluble thrombomodulin in patients with sepsis-associated coagulopathy (SCARLET): an updated meta-analysis. Crit Care. (2019) 23:302. 10.1186/s13054-019-2587-2 31488189 PMC6729086

[24] Abeyama K Stern DM Ito Y Kawahara K Yoshimoto Y Tanaka M. The N-terminal domain of thrombomodulin sequesters high-mobility group-B1 protein, a novel antiinflammatory mechanism. J Clin Invest. (2005) 115:1267–74. 10.1172/JCI22782 15841214 PMC1077171

[25] Egi M Ogura H Yatabe T Atagi K Inoue S Iba T. The Japanese clinical practice guidelines for management of sepsis and septic shock 2020 (J-SSCG 2020). Acute Med Surg. (2021) 8:e659. 10.1186/s40560-021-00555-7 34484801 PMC8390911

[26] Hayakawa M Kushimoto S Watanabe E Goto K Suzuki Y Kotani T. Pharmacokinetics of recombinant human soluble thrombomodulin in disseminated intravascular coagulation patients with acute renal dysfunction. Thromb Haemost. (2017) 117:851–9. 10.1160/TH16-07-0547 28229162 PMC5442600

[27] Watanabe E Yamazaki S Setoguchi D Sadahiro T Tateishi Y Suzuki T. Pharmacokinetics of standard- and reduced-dose recombinant human soluble thrombomodulin in patients with septic disseminated intravascular coagulation during continuous hemodiafiltration. Front Med. (2017) 4:15. 10.3389/fmed.2017.00015 28271063 PMC5318446

[28] Sugiyama S Hirota H Kimura R Kokubo Y Kawasaki T Suehisa E. Haplotype of thrombomodulin gene associated with plasma thrombomodulin level and deep vein thrombosis in the Japanese population. Thromb Res. (2007) 119:35–43. 10.1016/j.thromres.2005.12.012 16507317

[29] Navarro S Medina P Bonet E Corral J Martinez-Sales V Martos L. Association of the thrombomodulin gene c.1418C>T polymorphism with thrombomodulin levels and with venous thrombosis risk. Arterioscler Thromb Vasc Biol. (2013) 33:1435–40. 10.1161/ATVBAHA.113.301360 23520161

[30] Delvaeye M Noris M De Vriese A Esmon CT Esmon NL Ferrell G. Thrombomodulin mutations in atypical hemolytic-uremic syndrome. N Engl J Med. (2009) 361:345–57. 10.1056/NEJMoa0810739 19625716 PMC3530919

[31] Riedl M Fakhouri F Le Quintrec M Noone DG Jungraithmayr TC Fremeaux-Bacchi V. Spectrum of complement-mediated thrombotic microangiopathies: pathogenetic insights identifying novel treatment approaches. Semin Thromb Hemost. (2014) 40:444–64. 10.1055/s-0034-1376153 24911558

[32] Li Q Yang W Zhao K Sun X Bao L. Thrombomodulin gene polymorphism and the occurrence and prognostic value of sepsis acute kidney injury. Medicine. (2021) 100:e26293. 10.1097/MD.0000000000026293 34190147 PMC8257907

[33] Singer M Deutschman CS Seymour CW Shankar-Hari M Annane D Bauer M. The third international consensus definitions for sepsis and septic shock (Sepsis-3). JAMA. (2016) 315:801–10. 10.1001/jama.2016.0287 26903338 PMC4968574

[34] Seymour CW Kennedy JN Wang S Chang CH Elliott CF Xu Z. Derivation, validation, and potential treatment implications of novel clinical phenotypes for sepsis. JAMA. (2019) 321:2003–17. 10.1001/jama.2019.5791 31104070 PMC6537818

[35] Gando S Shiraishi A Yamakawa K Ogura H Saitoh D Fujishima S. Role of disseminated intravascular coagulation in severe sepsis. Thromb Res. (2019) 178:182–8. 10.1016/j.chest.2019.08.1025 31054468

[36] Reinhart K Bayer O Brunkhorst F Meisner M. Markers of endothelial damage in organ dysfunction and sepsis. Crit Care Med. (2002) 30(5 Suppl):S302–12. 10.1097/00003246-200205001-00021 12004252

